# Bats from the Pedra Branca Forest, Rio de Janeiro, Brazil

**DOI:** 10.3897/BDJ.9.e77400

**Published:** 2021-12-29

**Authors:** Jonatas Amorim Tavares, Roberto Leonan Morim Novaes, Iuri Veríssimo, Maria Alice do Amaral Kuzel, Sócrates Fraga da Costa-Neto, Caroline Lacorte Rangel, Mylena Borges, Helena Medrado, Bruno Alves, Renan de França Souza, Ana Carolina Duarte Pinto Menezes, Luis Fernando Menezes-Júnior, Daniela Dias, Cecilia Siliansky de Andreazzi, Rosana Gentile, Ricardo Moratelli

**Affiliations:** 1 Fiocruz Mata Atlântica, Fundação Oswaldo Cruz, Rio de Janeiro, Brazil Fiocruz Mata Atlântica, Fundação Oswaldo Cruz Rio de Janeiro Brazil; 2 Programa de Pós-Graduação em Biodiversidade e Saúde, Instituto Oswaldo Cruz, Fundação Oswaldo Cruz, Rio de Janeiro, Brazil Programa de Pós-Graduação em Biodiversidade e Saúde, Instituto Oswaldo Cruz, Fundação Oswaldo Cruz Rio de Janeiro Brazil; 3 Laboratório de Biologia de Tripanosomatídeos, Instituto Oswaldo Cruz, Rio de Janeiro, Brazil Laboratório de Biologia de Tripanosomatídeos, Instituto Oswaldo Cruz Rio de Janeiro Brazil; 4 Universidade Salgado de Oliveira, Rio de Janeiro, Brazil Universidade Salgado de Oliveira Rio de Janeiro Brazil; 5 Programa de Pós-Graduação em Biologia Animal, Instituto de Ciências Biológicas e da Saúde, Universidade Federal Rural do Rio de Janeiro, Rio de Janeiro, Brazil Programa de Pós-Graduação em Biologia Animal, Instituto de Ciências Biológicas e da Saúde, Universidade Federal Rural do Rio de Janeiro Rio de Janeiro Brazil; 6 Laboratório de Biologia e Parasitologia de Mamíferos Silvestres Reservatórios, Instituto Oswaldo Cruz, Fundação Oswaldo Cruz, Rio de Janeiro, Brazil Laboratório de Biologia e Parasitologia de Mamíferos Silvestres Reservatórios, Instituto Oswaldo Cruz, Fundação Oswaldo Cruz Rio de Janeiro Brazil

**Keywords:** bat survey, Chiroptera, urban forest, urban wildlife

## Abstract

The Pedra Branca Forest is located in a highly-urbanised region of the central portion of Rio de Janeiro City, comprises the largest urban forest on the continent and is isolated from other Atlantic Forest remnants. The local flora and fauna are protected by three conservation units (Pedra Branca State Park, Prainha Municipal Natural Park and Guaratiba State Biological Reserve) and one biological station (Fiocruz Atlantic Forest Biological Station—EFMA). Here, we provide an updated list of the bat fauna for the remnant. The results are based on samplings at EFMA and literature data from Pedra Branca State Park and Prainha Natural Park. The three sampling sites combined resulted in 31 species, 23 genera and four families. Phyllostomidae was the richest family with 24 species, followed by Vespertilionidae with five species (3%) and Molossidae and Noctilionidae with one species. The local bat fauna was predominantly composed of species with a broad geographic distribution.

## Introduction

Bats provide important ecosystem services as pollinators, seed dispersers and controllers of insect populations (Kunz et al. 2011). On the other hand, they have been implicated in many public health emergencies (e.g. SARS, Nipah, Hendra, Ebola and possibly COVID-19) as potential reservoirs of zoonotic deadly pathogens (Moratelli and Calisher 2015, Tang et al. 2020). Thus, understanding the structure of local bat faunas is essential for conservation programmes and the development of strategies in the One Health approach, particularly in areas under high anthropogenic pressure and social vulnerabilities, such as tropical forest remnants close to large urban centres (Lu et al. 2016; Beltz 2017; Kading and Kingston 2020).

The Brazilian Atlantic Forest is severely fragmented, particularly in large cities, such as Rio de Janeiro—the second largest City in Brazil, with more than 6.7 million people (IBGE 2020). The largest Atlantic Forest remnants in the City are in the massifs of Gericinó-Mendanha, Pedra Branca and Tijuca. These remnants are geographically isolated from each other, under severe anthropogenic pressure and mostly surrounded by an urban matrix. The Pedra Branca Forest covers most of the homonym Massif and extends to the adjacent lowlands on the eastern, western and southern slopes, comprising the largest urban forest in the world (Rocha et al. 2003). Most of the remnant is preserved by conservation units, amongst which Pedra Branca State Park (PEPB) is the largest conservation unit in the City of Rio de Janeiro, encompassing all areas above 100 m of elevation. The Fiocruz Atlantic Forest Biological Station (EFMA) is on the eastern slope of the Massif, encompassing lowland to submontane forests and overlapping partially with PEPB, in an area of high anthropogenic pressure, whose biological diversity, including mammals, is still little known compared to other localities (e.g. Tijuca, Reserva Biológica de Guapiaçu, Costa Verde Islands) in the State of Rio de Janeiro (Esberard 2003;Bolzan et al. 2010).

Here, we report the results of an extensive bat survey carried out at the Fiocruz Atlantic Forest Biological Station. As two other surveys have been conducted for bats in different regions of the remnant, we also provide an updated list of bats from the Pedra Branca Forest.

## Material and methods

### Study area

**Figure 1.**
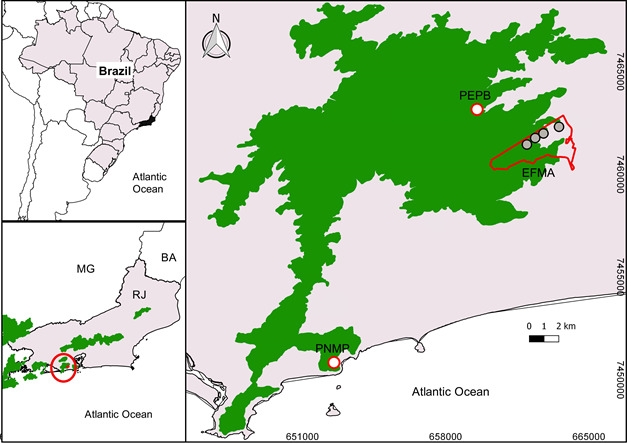
Pedra Branca Forest with indication of bat sampling sites (circles) at Fiocruz Atlantic Forest Biological Station (EFMA), Pedra Branca State Park (PEPB) and Prainha Natural Municipal Park (PNMP).

The Pedra Branca Forest (Fig. 1) comprises a locality of mountainous relief, with a maximum altitude of 1,024 m a.s.l. and is in a highly-urbanised region of the central portion of Rio de Janeiro City. The remnant is geographically isolated from other forest remnants and surrounded by an urban matrix, some plantations and shanty towns. Most of the territory is protected by conservation units (INEA 2013). Amongst them, Pedra Branca State Park (PEPB: 23°52'–23°04' S, 43°23'–43°32' W, ca. 12,400 hectares) encompasses all areas above 100 m of elevation. Other conservation units in the remnant and surroundings include Prainha Natural Park (PNMP: 23º01'–23º02' S, 43º30'–43º30' W, 147 ha) in the south; and Guaratiba State Biological Reserve (Reserva Biológica Estadual de Guaratiba [RBEG], 3,360 ha) in the west. The Fiocruz Atlantic Forest Biological Station (EFMA: 22°56'25" S; 43°24'18" W; 430 ha) is on the eastern slope of the Massif and overlaps partially with PEPB (261 ha), comprising a natural laboratory for biodiversity and health research.

Pedra Branca is predominantly classified as an ombrophilous dense forest (IBGE 2011), although there are also stretches of restinga shrubland in coastal areas within the PNMP. The cold and dry season extends from April–September and the warm rainy season extends from October–March. Köppen’s climate is Aw, with warm and rainy summers and dry winters, with annual mean temperatures ranging from 22–24°C and annual mean rainfall between 1300–1600 mm (Alvares et al. 2013). The Pedra Branca Forest has undergone an intense and complex history of land occupation and use. It started in the 16^th^ century with the agricultural cycles of sugarcane and coffee monocultures. Later in the 19^th^ century, there was intense use of natural areas for charcoal production. Since the 20^th^ century, the area has suffered unplanned urban occupation (Oliveira and Fernandez 2020). Consequently, the current vegetation cover is formed mainly by secondary forests in different stages of regeneration, including stretches of mature forest with a canopy reaching 20 m high, a diverse native flora and the presence of bromeliad epiphytes, orchids and adult palms (INEA 2013).

### Sampling and data survey

At EFMA, bat sampling was carried out for 55 nights using 10 mist-nets (polyester, 9 × 3 m, 20 mm mesh) that were placed in clearings in the vegetation, along trails, over water bodies and near flowering or fruiting plants (Kunz and Kurta 1988). Mist-nets were opened at sunset and closed after four hours. Sampling effort totalled 59,400 m².h. Captured animals were kept in cotton bags until being measured and identified. Most of the animals were released at the end of each sampling night. Some individuals were collected as a record of the species' existence and occurrence in the territory and for pathogen surveys. Specimens collected were deposited at the EFMA and animals were labelled FMA (Fiocruz Mata Atlântica; see Data resources). Individuals were identified by external and cranial traits, using identification characters described by Gardner (2008), Díaz et al. (2016) and Reis et al. (2017). *Myotis*, *Molossus* and *Lonchophylla* were identified according to Moratelli et al. (2011), Gregorin et al. (2011) and Dias et al. (2013), respectively. Nomenclature and classification followed Garbino et al. (2020).

This extensive list of bats from the Pedra Branca Forest was compiled, based on sampling efforts carried out by the Fiocruz Research Group from Oct 2013 to Dec 2017 in the EFMA and literature data from two other surveys carried out at the PEPB (Dias et al. 2002, Dias et al. 2003) and PNMP (Pinto 2008), totalling a sampling effort of 114,180 m².h. Other information on the occurrence of bat species for the Pedra Branca Forest was obtained from literature and used to complement the species list.

#### Data analyses

Bats were classified into trophic guilds following Kalko et al. (1996). Sampling effort was calculated following Straube and Bianconi (2002). Capture success was considered the ratio between the number of captures and the total effort employed. Estimation of maximum species richness was calculated using Jackknife-1 and Chao-1 in the software EstimateS 9.1 (Colwell 2013). Species accumulation curves were built for each sampling locality and for all localities combined to evaluate the adequacy of the sampling effort. The curves were built using the collector method, considering a descending order from the highest to the lowest value of bat species richness in the 'vegan' package for R software (Oksanen et al. 2018).

## Data resources

Voucher specimens (Suppl. material 1) collected at Fiocruz Atlantic Forest Biological Station (FMA), Rio de Janeiro, RJ, Brazil - *Carolliaperspicillata* (N = 10): FMA434, FMA435, FMA436, FMA438, FMA439, FMA440, FMA441, FMA443, FMA444, FMA445. *Tonatiabidens* (7): FMA437, FMA484, FMA488, FMA1570, FMA1576, FMA1584, FMA1656. *Micronycterismicrotis* (4): FMA446, FMA535, FMA561, FMA1517. *Desmodusrotundus* (10): FMA447, FMA448, FMA449, FMA453, FMA454, FMA455, FMA463, FMA472, FMA482, FMA483. *Glossophagasoricina* (7): FMA450, FMA467, FMA480, FMA503, FMA511, FMA518, FMA1521. *Myotisnigricans* (12): FMA452, FMA462, FMA476, FMA1557, FMA1562, FMA1564, FMA1566, FMA1569, FMA1574, FMA1575, FMA1606, FMA1644. *Artibeusfimbriatus* (7): FMA456, FMA485, FMA499, FMA515, FMA524, FMA897, FMA1578. *Artibeuslituratus* (12): FMA457, FMA464, FMA470, FMA471, FMA473, FMA477, FMA479, FMA481, FMA493, FMA498, FMA504, FMA514. *Sturniralilium* (8): FMA458, FMA461, FMA506, FMA516, FMA525, FMA532, FMA539, FMA1502. *Platyrrhinusrecifinus* (2): FMA475, FMA1540. *Vampyressapusilla* (9): FMA489, FMA495, FMA500, FMA527, FMA533, FMA1518, FMA1616, FMA1652, FMA1653. *Artibeusobscurus* (7): FMA490, FMA529, FMA1508, FMA1509, FMA1550, FMA1581, FMA1605. *Platyrrhinuslineatus* (1): FMA565. *Mimonbennetti* (2): FMA491, FMA501. *Phyllostomushastatus* (6): FMA492, FMA494, FMA558, FMA1511, FMA1551, FMA1637. *Myotisriparius* (6): FMA496, FMA1556, FMA1558, FMA1559, FMA1560, FMA1561. *Glyphonycterissylvestris* (1): FMA502. *Sturniratildae* (4): FMA507, FMA509, FMA554, FMA1531. *Micronycterisminuta* (3): FMA519, FMA541, FMA1610. *Lonchophyllaperacchii* (3): FMA526, FMA891, FMA1631. *Anouracaudifer* (3): FMA555, FMA900, FMA1659. *Molossusmolossus* (2): FMA1628, FMA1629.

## Results

### Bats from EFMA

A total of 558 individuals were captured at EFMA (success of 0.009 captures/m².h), representing 25 species from three families (Table 1). Phyllostomidae was the most abundant and richest family, representing 95% of the total sampling (530 individuals) and 20 species. Vespertilionidae was represented by 26 individuals (4.5% of sampling) and four species and Molossidae was represented by two individuals (0.5%) and one species. *Artibeuslituratus* (N = 217), *Carolliaperspicillata* (N = 153) and *Desmodusrotundus* (N = 42) were the most abundant species.

### Bats from the Pedra Branca Forest

Our sampling site, combined with the two extra localities, resulted in an effort of 123 sampling nights and 114,180 m².h, with 1,644 individuals captured (Table 1). The capture success combined was 0.014, varying per locality from 0.009 to 0.025 captures/m².h (Table 2). In total, 29 species from 22 genera and three families were recorded at the three study sites (Table 1). Phyllostomidae was the most sampled and richest family, with 1,582 individuals representing 24 species (96% of the total sampling); followed by Vespertilionidae, with 47 individuals and five species (3%); and Molossidae, with 15 individuals and one species (1%; Table 1). *Artibeuslituratus* (N = 596, 37%), *Carolliaperspicillata* (N = 350, 22%) and *Artibeusfimbriatus* (N = 239, 15%) were the most abundant species in the Pedra Branca Forest. Silva et al. (2019) reported the occurrence of *Anourageoffroyi* (Phyllostomidae) and *Noctilioleporinus* (Noctilionidae) in the region. Thus, 31 species have been recorded for the Pedra Branca Forest so far. Amongst them, 16 were registered at EFMA, PEPB and PNMP. Four species (*D.ecaudata*, *C.auritus*, *A.geoffroyi* and *N.leporinus*) were found only at PEPB, four only at EFMA (*M.microtis*, *S.tildae*, *M.izecksohni* and *M.riparius*) and one (*H.velatus*) only at PNMP.

**Figure 2.**
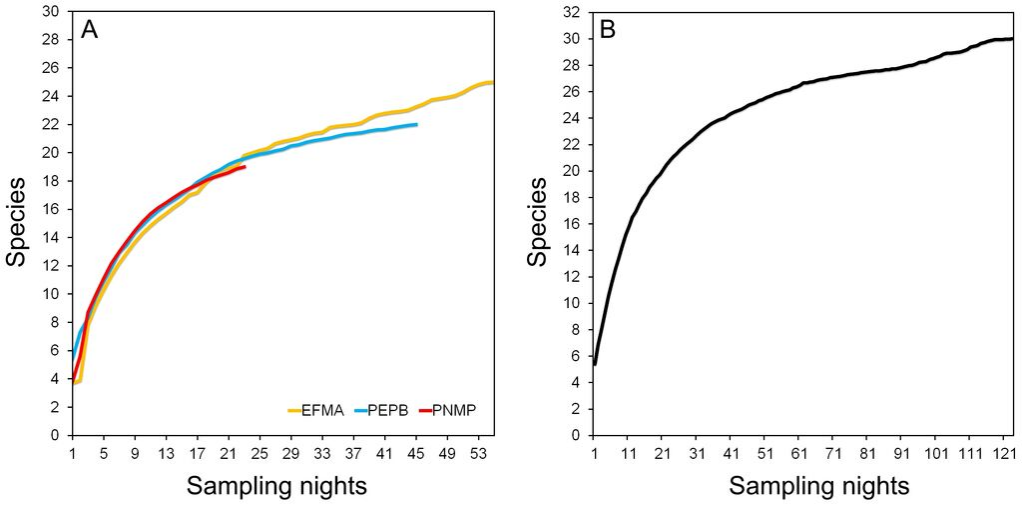
Species accumulation curves for each sampled area (**A**) and for the three studies gathered (**B**) in Pedra Branca Forest, Rio de Janeiro, RJ, Brazil.

Species accumulation curves did not show stabilisation, neither for each locality (Fig. 2A) nor for all localities combined (Fig. 2B), indicating insufficient sampling. In addition, Jackknife-1 and Chao-1 estimators of species richness indicated that our samplings corresponded to 85–90% of the expected species for each locality and for all localities combined (Table 3).

## Discussion

### Species richness and composition

The three localities in the Pedra Branca remnant altogether revealed 31 species of bats, which represents 40% of the 80 species reported for the State of Rio de Janeiro (Dias et al. 2003, Peracchi and Nogueira 2010, Moratelli et al. 2011, Dias et al. 2013, Delciellos et al. 2018) and 31% of the 98 species reported for the Atlantic Forest in Brazil (Muylaert et al. 2017). The phyllostomids, *Artibeuslituratus*, *A.fimbriatus* and *Carolliaperspicillata*, were the most abundant species, comprising 73% of the sampling. These three species are amongst the most abundant in Atlantic Forest surveys (see Faria 1997, Faria 2006, Souza et al. 2014, Muylaert et al. 2017, Novaes et al. 2017). The species accumulation curves corroborated the results of the estimated species richness, indicating that there might be species not sampled in the study area and that the number of species may increase with more sampling effort.

Dias et al. (2002) reported the occurrence of *Lonchophyllamordax* and *Lonchophyllabokermanni* at Pedra Branca Forest. However, a subsequent review of *Lonchophylla* from South-eastern Brazil by Dias et al. (2013) re-assigned those specimens to *L.peracchii*. Currently, *Lonchophyllamordax* seems to be restricted to the Caatinga of Northeast Brazil, whereas *L.bokermanni* is restricted to the semi-deciduous forest and savannah areas of the Espinhaço Mountain Range, with no records of these two species for the Atlantic Forest of Rio de Janeiro (Dias et al. 2013, Moratelli and Dias 2015, Cláudio et al. 2018).

The record of *Micronycterismegalotis* for the Pedra Branca Forest by Dias et al. (2002) represents misidentifications of *Micronycterismicrotis* (see Dias and Peracchi 2008). Silva et al. (2019) reported the occurrence of *Artibeusplanirostris* at the Pedra Branca Forest. However, an unpublished revision of *Artibeus* specimens from the Atlantic Forest of Rio de Janeiro (including material from Pedra Branca) conducted by one of us (D. Dias) did not find evidence of the occurrence of the species in the State.

### Future directions

In general, bat surveys in the Atlantic Forest are based on ground-level mist-nets only. Although this method is widely used throughout the Neotropical Region (Kunz and Kurta 1988, Trevelin et al. 2017), it has selective efficiency. Some phyllostomid bats (particularly Stenodermatinae and Carolliinae) are more easily captured in ground-level mist-nets than other taxa (Nowak 1994). This explains the high species richness of this family in the study area, although phyllostomids represent less than 50% of all known species for Rio de Janeiro (Peracchi and Nogueira 2010). Bats from other families, especially Emballonuridae, Molossidae and Vespertilionidae, are more difficult to capture because they are generally aerial insectivores that capture their prey during flight in open areas or above the tree canopy (Nowak 1994, Marques et al. 2015). Furthermore, these bats can detect and avoid nets easier than others due to their more efficient echolocation and great manoeuvrability (Marques et al. 2015). These additional methods include the use of canopy mist nets and bioacoustic surveys. These methods have been shown to be especially effective for detecting aerial insectivores in tropical forests (Marques et al. 2015, Hintze et al. 2016, Gregorin et al. 2016). As an example, an extensive sampling was carried out over water bodies in the Tijuca Forest, which favoured the record of six species of molosids (Esberard 2003) against only one molosid in Pedra Branca, which were mainly collected along existing trails.

We expect an increase in the species list for the Pedra Branca Forest by sampling in localities not previously surveyed and using different and complementary methods. Considering that the study area is under high anthropogenic pressure, is located in an urban area with the second largest population density in Brazil and that bats are one of the most important groups to host zoonotic pathogens, the high species richness found highlights the importance of long-term monitoring in these areas within the One Health approach.

## Supplementary Material

D2FC717A-A48A-5A8D-8FC2-A8150D6CBABC 10.3897/BDJ.9.e77400.suppl1 Supplementary material 1 Linked data table of Bats of the Pedra Branca Forest Data type Occurrence records File: oo_624891.xlsx https://binary.pensoft.net/file/624891 Amorim et al.

## Figures and Tables

**Table 1.** Checklist of bat species from the Pedra Branca Forest, Rio de Janeiro, Brazil, including information on number of captures by locality and diet. Localities include Fiocruz Atlantic Forest Biological Station (EFMA; present study), Pedra Branca State Park (PEPB; Dias et al. 2002) and Prainha Municipal Natural Park (PNMP; Pinto 2008). Species, whose presence in the locality is marked with an “X”, were obtained from Silva et al. (2019).

**Taxon**	**Diet**	**Individuals per locality**			**Total**
		**EFMA**	**PEPB**	**PNMP**	
**Phyllostomidae, Micronycterinae**					
*Micronycterismicrotis*	Gleaning insectivore	2	1	0	3
*Micronycetrisminuta*	Gleaning insectivore	7	1	3	12
**Phyllostomidae, Desmodontinae**					
*Desmodusrotundus*	Sanguivore	42	41	2	85
*Diphyllaecaudata*	Sanguivore	0	4	0	4
**Phyllostomidae, Phyllostominae**					
*Chrotopterusauritus*	Carnivore	0	3	0	3
*Mimonbennettii*	Gleaning insectivore	2	1	0	3
*Phyllostomushastatus*	Omnivore	10	6	0	16
*Tonatiabidens*	Omnivore	8	2	3	13
*Trachopscirrhosus*	Carnivore	1	0	3	4
**Phyllostomidae, Glossophaginae**					
*Anouracaudifer*	Nectarivore	4	11	9	24
*Anourageoffroyi*	Nectarivore	-	X	-	X
*Glossophagasoricina*	Omnivore	9	17	18	44
**Phyllostomidae, Lonchophyllinae**					
*Lonchophyllaperacchii*	Nectarivore	3	3	0	6
**Phyllostomidae, Carolliinae**					
*Carolliaperspicillata*	Frugivore	153	100	96	350
**Phyllostomidae, Glyphonycterinae**					
*Glyphonycteryssylvestris*	Gleaning insectivore	1	1	0	1
**Phyllostomidae, Stenodermatinae**					
*Artibeusfimbriatus*	Frugivore	25	139	75	239
*Artibeuslituratus*	Frugivore	217	265	114	596
*Artibeusobscurus*	Frugivore	10	20	23	53
*Chirodermadoriae*	Frugivore	0	5	3	8
*Platyrrhinuslineatus*	Frugivore	1	5	23	29
*Platyrrhinusrecifinus*	Frugivore	2	2	6	10
*Sturniralilium*	Frugivore	20	27	3	50
*Sturniratildae*	Frugivore	4	0	0	4
*Vampyressapusilla*	Frugivore	9	7	6	22
**Noctilionidae**					
*Noctilioleporinus*	Piscivore	-	X	-	X
**Molossidae**					
*Molossusmolossus*	Aerial insectivore	2	8	5	15
**Vespertilionidae**					
**Vespertilioninae**					
*Eptesicusbrasiliensis*	Aerial insectivore	1	1	2	3
*Histiotusvelatus*	Aerial insectivore	0	0	2	2
**Myotinae**					
*Myotisizecksohni*	Aerial insectivore	2	0	0	2
*Myotisnigricans*	Aerial insectivore	13	11	6	32
*Myotisriparius*	Aerial insectivore	10	0	0	10
**Total of captures**		**558**	**681**	**402**	**1,644**

**Table 2.** Species richness, capture effort and capture success of bats in three surveys in Pedra Branca Forest, Rio de Janeiro, RJ, Brazil. Localities: EFMA = Fiocruz Atlantic Forest Biological Station (present study); PEPB = Pedra Branca State Park (Dias et al. 2002) and PNMP = Prainha Municipal Natural Park (Pinto 2008).

**Localities**	**Sampling nights**	**Captures (N)**	**Species richness**	**Sampling** **Effort (m².h)**	**Capture success**	
						EFMA	55	558	25	59,400	0.009	
PEPB	45	681	24	38,880	0.017	
PNMP	23	402	19	15,900	0.025	
**Total**	**123**	**1,639**	**31**	**114,180**	**0.014**	

**Table 3.** Estimated species richness of bats using Jackknife1 and Chao1 indices for each sampled area and for the three studies gathered in Pedra Branca Forest, Rio de Janeiro, RJ, Brazil.

**Localities**	**N Species**	**Jackknife-1**	**Chao-1**
EFMA	25	28	26
PEPB	24	26	27
PNMP	19	22	20
**Total**	**31**	**33**	**30**

## Hosting institution

Fundação Oswaldo Cruz - Fiocruz
